# Newtonian-Type Adaptive Filtering Based on the Maximum Correntropy Criterion

**DOI:** 10.3390/e22090922

**Published:** 2020-08-22

**Authors:** Pengcheng Yue, Hua Qu, Jihong Zhao, Meng Wang

**Affiliations:** 1 School of Electronic and Information Engineering, Xi’an Jiaotong University, Xi’an 710049, China; qh@mail.xjtu.edu.cn (H.Q.); zhaojihong@mail.xjtu.edu.cn (J.Z.); 2 School of Software Engineering, Xi’an Jiaotong University, Xi’an 710049, China; avictor.wally@stu.xjtu.edu.cn; 3 School of Communications and Information Engineering, Xi’an University of Posts and Telecommunications, Xi’an 710061, China

**Keywords:** Newtonian method, robust adaptive filter, maximum correntropy criterion, steady-state performance analysis, acoustic echo cancellation

## Abstract

This paper provides a novel Newtonian-type optimization method for robust adaptive filtering inspired by information theory learning. With the traditional minimum mean square error (MMSE) criterion replaced by criteria like the maximum correntropy criterion (MCC) or generalized maximum correntropy criterion (GMCC), adaptive filters assign less emphasis on the outlier data, thus become more robust against impulsive noises. The optimization methods adopted in current MCC-based LMS-type and RLS-type adaptive filters are gradient descent method and fixed point iteration, respectively. However, in this paper, a Newtonian-type method is introduced as a novel method for enhancing the existing body of knowledge of MCC-based adaptive filtering and providing a fast convergence rate. Theoretical analysis of the steady-state performance of the algorithm is carried out and verified by simulations. The experimental results show that, compared to the conventional MCC adaptive filter, the MCC-based Newtonian-type method converges faster and still maintains a good steady-state performance under impulsive noise. The practicability of the algorithm is also verified in the experiment of acoustic echo cancellation.

## 1. Introduction

Adaptive filtering is widely used in many areas including system identification, channel equalization, interference cancelling, acoustic echo cancellation(AEC), etc. [1,2,3,4,5]. Traditional adaptive filtering methods based on minimum mean square error (MMSE) criterion perform well in the presence of Gaussian noise, and the optimization methods adopted are mostly least mean square (LMS)-type or recursive least square (RLS)-type [6]. LMS-type adaptive filtering uses gradient descent characterized with a low convergence speed and a very low arithmetic complexity, while RLS-type adaptive filtering, free from the selection problem of the optimal step size, converges much faster at the cost of higher complexity, and is afflicted with stability problems caused by error propagation and unregulated matrix inversion [7]. Kalman filter is also an important optimization method in state estimation [6].

MMSE criterion-based adaptive filtering suffers from impulsive noise for its sensitivity to large outliers. To deal with this problem, robust adaptive filtering has been researched extensively. A popular robust solution is to change the MMSE criterion to other criteria that suppress impulsive noise, such as Huber loss [8], least p-norm criterion [9], etc. In recent years, information theory learning (ITL) was found suitable to deal with non-Gaussian noises [10,11,12,13,14]. Inspired by ITL, maximum correntropy criterion (MCC) or generalized maximum correntropy criterion (GMCC)-based adaptive filtering was studied [15,16,17,18,19,20]. Most of the aforementioned robust algorithms were LMS-like [21,22] or RLS like [23,24,25], which is to say that the optimization methods used were limited to gradient descent and fixed point iteration [26]. MCC-based Kalman filter as an optimization method of state estimation was studied in [27]. Ref. [28] implied that a Newtonian algorithm could be utilized in the MCC state estimation, as correntropy is a differentiable function. However, different methods such as Newton’s method and all its derivative algorithms that converge faster than gradient descent are seldom considered in MCC-based adaptive filtering. As an inspiring attempt to enrich the optimization methods, ref. [29,30] proposed a correntropy-based Levenberg–Marquardt algorithm that converges faster than maximum correntropy-based gradient descent algorithm and performs well dealing with heavy tailed non-Gaussian noise. This work revealed the potential adoption of more optimization methods except gradient descent and fixed point iteration. Especially, Newtonian-type optimization methods of MCC-based robust adaptive filtering are far from complete and still need to be improved.

Adaptive filtering based on Newtonian or quasi-Newtonian methods was proved to be serviceable for its fast convergence rate [31,32,33,34,35,36]. Adaptive filtering based on Newtonian methods, known as LMS-Newton, models the input sequence as an autoregressive (AR) process, and usually focuses on the acceleration of the estimate of the input autocorrelation matrix [32,33]. Adaptive filtering based on quasi Newtonian methods, known as quasi-Newtonian adaptive filtering, usually updates the approximation of the Newtonian direction (typically the inverse Hessian matrix) by formulas similar to that of BFGS [34,37]. Adaptive filtering based on the Gauss–Newton method or Levenberg–Marquardt (LM) method also put forward approximations of the Hessian matrix that are easy to compute using the Jacobian matrix [35]. Moreover, there are many methods adopted to enhance the robustness of Newtonian-type adaptive filtering [31,34,36]. Reference [36] proposed a robust algorithm and revealed that the weighting function related to the cost function in the algorithm is the key that ensures the robustness. Inspired by that, we adopt LMS-Newton to solve the optimization of the cost function based on MCC, and we call this the MCC-Newton adaptive filtering method, which enhances the existing body of knowledge in MCC-based adaptive filtering. The proposal is in the category of the most commonly used linear transversal filter.

The main contributions of this paper are as follows: (1) the Newtonian-type optimization method is introduced in MCC-based adaptive filter and the recursive updating equation of the impulse response is derived. (2) The steady-state performance analysis is discussed theoretically and compared with that in experiments. According to the theoretical analysis, the guideline of parameter selection of the algorithm is provided. (3) The algorithm is applied in system identification and acoustic echo cancellation in experiments to verify the practicability.

The paper is divided into five parts. Section 2 presents the conventional Newtonian-type adaptive filter based on the MMSE criterion and introduces the MCC. Section 3 proposes the Newtonian-type adaptive filter based on MCC and gives the recursive solution of the impulsive response. The complexity of the algorithm is also compared with that of the other algorithms. Section 4 analyzes the steady-state performance of the algorithm theoretically. The experiments verifying the steady-state performance discussion is displayed in Section 5, and there are also experiments showing that the proposed algorithm is robust in the presence of impulsive noise and converges faster than the gradient descent-based adaptive filter algorithms. Besides, the experiment of acoustic echo cancellation verifies the practicability of the algorithm. Section 6 gives the conclusion of the paper.

## 2. Preliminaries

### 2.1. Conventional Newtonian-Type Adaptive Filtering

The adaptive filter update equation of the conventional LMS-Newton algorithm is implemented as in [33,36]:

(1) e(n)=d(n)−y(n)

(2) $$W\left(n+1\right)=W\left(n\right)+\mu e\left(n\right){\hat{R}}^{-1}\left(n\right)X\left(n\right),$$

where e(n) is the estimation error at time point *n*, d(n) is the observed system output, and the linear transversal filter estimated output $y\left(n\right)=X^{T}\left(n\right)W\left(n\right)$ is the filter parameter W(n) multiplying the input X. μ is the step size, and $\hat{R}$ is the estimated autocorrelation matrix of X(n) which is assumed to be known in the ideal LMS-Newton algorithm. Note that when $\hat{R}$ equals to the identity matrix I, (2) becomes the equation of the conventional LMS algorithm. The ideal algorithm is easy to analyze theoretically, but it is considered impractical for the computational complexity.

In practical articles considering the application of the LMS-Newton method in acoustic echo cancellation, the input X(n) is modeled as an autoregressive (AR) process in which the order is much smaller than the length of the filter [33,38,39]. According to the characteristics of AR modeling, there are many efficient ways to simplify the computation and updating of ${\hat{R}}^{-1}\left(n\right)X\left(n\right)$. Some of the articles estimate ${\hat{R}}^{-1}\left(n\right)$ first and then perform the matrix multiplication [38,39], while other articles directly compute the vector ${\hat{R}}^{-1}\left(n\right)X\left(n\right)$ without the estimation of R(n) [33]. Owing to the efficient updating of ${\hat{R}}^{-1}\left(n\right)X\left(n\right)$, the modified practical LMS-Newton algorithms could keep very small computational complexity, which is approximately equal to that of the conventional LMS algorithm, adding a negligible updating operation of ${\hat{R}}^{-1}\left(n\right)X\left(n\right)$. Practical Newtonian-type adaptive filtering has the potential to converge as fast as RLS-type adaptive filtering while maintaining a low computational complexity.

### 2.2. Maximum Correntropy Criterion

Conventional Newtonian-type adaptive filtering derives the equation of the adaptive filter parameter according to the MMSE criterion, which performs well under Gaussian noise but suffers from non-Gaussian noise. In this paper, MCC is introduced to enhance the robustness of the Newtonian-type adaptive filtering. Correntropy in ITL is used to measure the similarity of two random variables, and is defined as [10]:

(3) $$V\left(X,Y\right)=E\left[\kappa \left(X,Y\right)\right]=\int \kappa \left(x,y\right)dF_{XY}\left(x,y\right),$$

where X,Y denote two random variables; E[·] is the expectation operator; $F_{XY}\left(x,y\right)$ represents the joint distribution function of (X,Y); and κ(·) stands for a Mercer kernel which is in general the Gaussian kernel defined as:

(4) $$\kappa_{\beta }\left(x,y\right)=G_{\beta }\left(x-y\right)=\frac{1}{\sqrt{2\pi }\beta }\exp\left(-\frac{{\left|x-y\right|}^{2}}{2\beta^{2}}\right),$$

where β is the Gaussian kernel width, and $1/\sqrt{2\pi \beta }$ is the normalization parameter.

In practical adaptive filtering, the available data ${\left\{x_{n},y_{n}\right\}}_{n=1}^{N}$ of X,Y are discrete and the joint distribution $F_{XY}\left(x,y\right)$ can be estimated by the Parzen kernel estimator as:

(5) $${\hat{V}}_{N}^{\beta }\left(X,Y\right)=\frac{1}{N}\sum_{n=1}^{N}\kappa_{\beta }\left(x_{n}-y_{n}\right).$$

### 2.3. Comparison of Different Criteria

This section presents the characteristic of robustness of MCC to outliers and gives a formal motivation for the use of MCC. The loss functions of different criteria are the metrics of the estimation error *e*. We compare the correntropy induced loss function defined in (4) with the MSE loss function and its general version, Lp norm loss function.

The MSE loss, in other words, L2 loss is defined as:

(6) $$L_{MSE}={\left|e\right|}^{2}.$$

The Lp norm loss can be defined as

(7) $$L_{Lp}={\left|e\right|}^{p},$$

where *p* usually satisfies 1<p<2. When p=2, Lp norm loss becomes L2 loss.

Note that the correntropy induced loss function (C-loss) defined in (4) is a concave function. So in MCC, we try to find the maximum of the cost function to reduce the estimation error. However, we usually wish the loss function to be convex, so that the function value will get larger as the absolute value of the error increases. So we used a modified version of C-loss [40] to compare with the other loss functions. The modified C-loss is convex and can be defined as:

(8) $$L_{C}=\frac{1}{\sqrt{2\pi }\beta }\left(1-\exp\left(-\frac{{\left|e\right|}^{2}}{2\beta^{2}}\right)\right).$$

**Figure 1:**
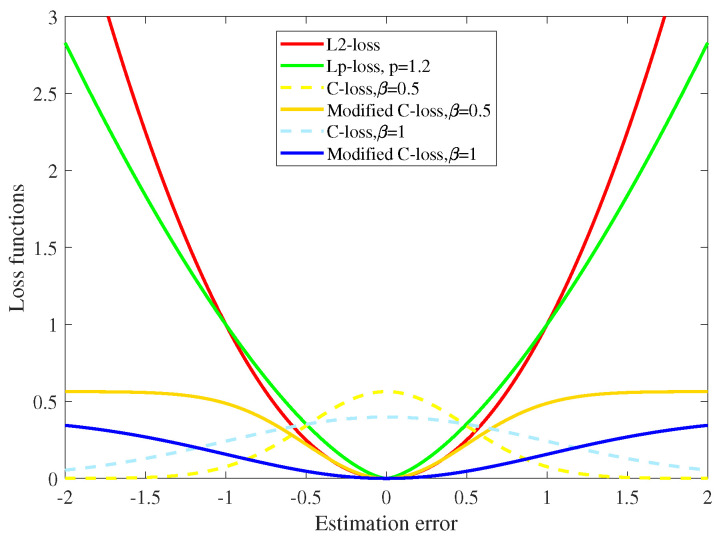
Comparision of different loss functions.

Figure 1 shows the loss function of different criteria. For each convex loss function, the function value increases with the growth of the absolute value of the estimation error. When an outlier appears, the value of L2-loss and Lp-loss becomes very large, but the value of C-loss remains relatively stable. Hence one can conclude that compared with the conventional loss functions, C-loss is robust against outliers.

## 3. A Newtonian-Type Adaptive Filtering Based on MCC

In our robust Newtonian-type adaptive filtering, MCC is used to construct the cost function J=E[p(e(n))], in which p(e(n)) is the loss function of the error e(n) at time point *n*, and can be written as $G_{\beta }\left(e\left(n\right)\right)$ in MCC instead of $e^{2}\left(n\right)$, the L2 loss in MMSE criterion. To calculate the optimal estimation of the adaptive filter parameter W(n), the gradient of the cost function with respect to W(n) is set to zero and we can derive that

(9) $$E\left[G_{\beta }\left(d-y\right)\left(d-y\right)X\right]=0$$

(10) $$E\left[G_{\beta }\left(e\right)\left(d-X^{T}W\right)X\right]=0$$

and

(11) $$E\left[G_{\beta }\left(e\right)XX^{T}\right]\cdot W=E\left[G_{\beta }\left(e\right)d\cdot X\right].$$

**Figure 2:**
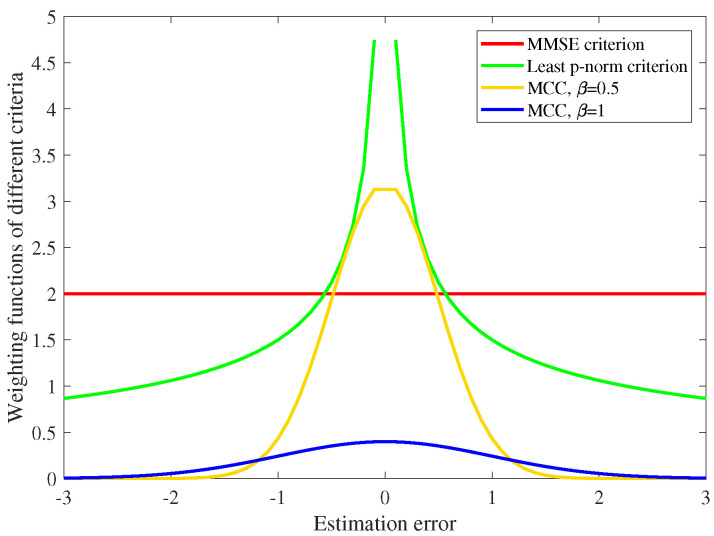
Weighting functions of different criteria.

In adaptive filtering, the data are discrete and expectation operators can be presented using their sample estimator:

(12) $$R_{MCC}\cdot W=P_{MCC},$$

where $R_{MCC}=\sum_{n=1}^{N}q\left(e(n)\right)X\left(n\right)X{\left(n\right)}^{T}$ and $P_{MCC}=\sum_{n=1}^{N}q\left(e(n)\right)d\left(n\right)\cdot X\left(n\right)$. q(e(n)) is the weighting function of RLS-type MCC-based adaptive filtering that values how much the *n*-th sample data influences the filtering. In Newtonian-type and RLS-type adaptive filtering, the weighting function can be calculated by [19,36]:

(13) $${q\left(e\left(i\right)\right)=|p\left(e\left(i\right)\right)}^{\prime }/e\left(i\right)|,$$

where p(e(n)) is the loss function or the error measurement. One can calculate that the error measurement and the weighting function are coincidentally $G_{\beta }\left(e\left(n\right)\right)$ in terms of MCC. In MMSE criterion, p(e(n)) is set to $e^{2}\left(n\right)$, then q(e(i)) equals to constant 2 and (12) will become the conventional RLS solution. The weighting functions of different criteria are displayed in Figure 2, which shows that MCC assigns very little weight on sample data that cause large estimation error compared with MMSE criterion and least p-norm criterion. Therefore, MCC is robust against outliers and able to diminish the impact of impulsive noises.

Similar to the derivation process of the conventional LMS-Newton, the gradient of W can be calculated as

(14) $$\nabla_{MCC}=\frac{\partial J_{MCC}\left(n\right)}{\partial w}=2R_{MCC}\cdot W-2P_{MCC}.$$

Adding a multiplier $1/2*R_{MCC}^{-1}$ to both sides of the formula, one can get

(15) $$W_{MCC}^{*}=W-\frac{1}{2}R_{MCC}^{-1}\nabla_{MCC}.$$

Meanwhile, from (10) we can derive the gradient $\nabla_{MCC}$ as

(16) $$\nabla_{MCC}=\frac{1}{N}\sum_{n=1}^{N}G_{\beta }\left(e\left(n\right)\right)\left(e(n)\right)X\left(n\right)$$

where we retain the information of all moments but not take the instantaneous gradient as a substitute like other algorithms.

Combining (15) and (16) one can derive the expression of the MCC-based Newtonian-type adaptive filtering as:

(17) $$W_{MCC}\left(n+1\right)=W_{MCC}\left(n\right)+\mu \cdot R_{MCC}^{-1}\left(n\right)F_{MCC}\left(n\right)X_{MCC}\left(n\right),$$

where $F_{MCC}\left(n\right)$ is a vector in which each element is $G_{\beta }\left(e\left(n\right)\right)\left(e(n)\right)$ of each time point, and $X_{MCC}\left(n\right)$ is a matrix composed by vectors from X(1) to X(n). A step size μ is also added in the recursive equation to enhance the flexibility of the algorithm. Note that the MCC-Newton algorithm of this paper is different from that of [36], because the error vector and the input matrix in (17) contain the information of all the previous time points. In practical implementation of calculating the gradient according to (16), a forgetting factor and a sliding window could be also adopted to enhance the tracking ability and reduce the computational complexity, just like in the sliding-exponential window RLS adaptive filtering or sliding window LMS algorithm [41,42].

## 4. Steady-State Performance Analysis

The steady-state performance analysis [21,22,43,44] of adaptive filtering is a theoretical foundation and gives the guideline for the parameter selection.

Firstly, the assumptions used throughout the analysis are given as follows.

A1: The additive noise sequence v(n) with variance $\sigma_{v}^{2}$ is independent and identically distributed (i.i.d.), and is independent of the input sequence X(n).

A2: The filter is enough long so that the a priori error $e_{a}\left(n\right)$ is zero-mean Gaussian and independent of the background noise v(n).

We define the steady-state MSE as:

(18) $$MSE=\lim_{n\to \infty }E\left[e^{2}\left(n\right)\right].$$

The steady-state excess mean square error (EMSE) can be presented as:

(19) $$EMSE=\lim_{n\to \infty }E\left[e_{a}^{2}\left(n\right)\right].$$

So

(20) $$MSE=EMSE+\sigma_{v}^{2}.$$

In our theoretical analysis of the steady-state behavior of the algorithm, the desired output of the unknown system d(n) can be presented as:

(21) $$d\left(n\right)=W_{o}^{T}X\left(n\right)+v\left(n\right),$$

where $W_{o}$ is the optimal impulsive response vector of the adaptive filter which could not be measured directly, and v(n) is the additive noise at time point *n*.

The weight error vector is presented as:

(22) $$\tilde{W}\left(n\right)=W_{0}-W\left(n\right).$$

To simplify the structure of the discussed expressions, we assume that the width of the sliding window is 1, then (17) becomes

(23) $$W\left(n+1\right)=W\left(n\right)+\mu \cdot G_{\beta }\left(e\left(n\right)\right)e\left(n\right)R^{-1}\left(n\right)X\left(n\right).$$

Combining (23) and (22), one can obtain:

(24) $$\tilde{W}\left(n+1\right)=\tilde{W}\left(n\right)-\mu \cdot G_{\beta }\left(e\left(n\right)\right)e\left(n\right)R^{-1}\left(n\right)X\left(n\right).$$

We define prior and posteriori errors as

(25) $$\begin{array}{cc} \; & e_{a}\left(n\right)=\tilde{W}{\left(n\right)}^{T}X\left(n\right)\\ \; & e_{p}\left(n\right)=\tilde{W}{\left(n+1\right)}^{T}X\left(n\right) \end{array}$$

Premultiplying $X^{T}\left(n\right)$ on both sides of (24), one can get

(26) $$e_{p}\left(n\right)=e_{a}\left(n\right)-\mu \cdot G_{\beta }\left(e\left(n\right)\right)e\left(n\right)X{\left(n\right)}^{T}R^{-1}\left(n\right)X\left(n\right).$$

As a stable algorithm, the MCC-Newton converges to the steady-state when time point *n* comes to a very large number. So the weight error vector satisfies [44]:

(27) $$\lim_{n\to \infty }E\left[∥\tilde{W}{\left(n\right)∥}^{2}\right]=\lim_{n\to \infty }E\left[∥\tilde{W}{\left(n+1\right)∥}^{2}\right]$$

Combining (25) and (27), one can derive:

(28) $$\lim_{n\to \infty }E\left[e_{p}{\left(n\right)}^{2}\right]=\lim_{n\to \infty }E\left[e_{a}{\left(n\right)}^{2}\right].$$

At the steady-state, the time index *n* can be omitted for brevity since the distributions of the input and error signals are independent with *n*. We also omit the limit operators of the formulas and rewrite (28) as:

(29) $$E\left(e_{a}^{2}\right)=E\left(e_{p}^{2}\right).$$

Substituting (26) into (29), one can derive that

(30) $$\begin{array}{c} E\left(e_{a}^{2}\right)=E\left(e_{a}^{2}\right)-2E\left(e_{a}\mu G_{\beta }\left(e\right)e\cdot X^{T}R^{-1}X\right)+E\left({\left(X^{T}R^{-1}X\right)}^{2}\cdot {\left(\mu G_{\beta }\left(e\right)e\right)}^{2}\right) \end{array}$$

and

(31) $$2E\left(e_{a}\mu G_{\beta }\left(e\right)e\right)=E\left(X^{T}R^{-1}X\cdot {\left(\mu G_{\beta }\left(e\right)e\right)}^{2}\right).$$

$X^{T}R^{-1}X$ is independent from $\mu G_{\beta }\left(e\right)e$, so that $E\left(X^{T}R^{-1}X\cdot {\left(\mu G_{\beta }\left(e\right)e\right)}^{2}\right)=E\left(X^{T}R^{-1}X\right)\cdot E\left({\left(\mu G_{\beta }\left(e\right)e\right)}^{2}\right)$.

Assume that $f\left(e\right)=(\mu G_{\beta }\left(e\right)e)$, then (31) becomes

(32) $$2E\left(e_{a}\cdot f\left(e\right)\right)=E\left(X^{T}R^{-1}X\right)E\left(f{\left(e\right)}^{2}\right).$$

It is known that $e\left(n\right)=e_{a}\left(n\right)+v\left(n\right)$. The simplification of (32) needs the Taylor expansion of f(e) [21].

(33) $$f\left(e\right)=f\left(v+e_{a}\right)=f\left(v\right)+f^{\prime }\left(v\right)e_{a}+\frac{1}{2}f^{\prime \prime }\left(v\right)e_{a}^{2}+o\left(e_{a}\right).$$

Then one can derive the following expressions:

(34) $$E\left[e_{a}f\left(e\right)\right]=E\left[\left(e_{a}f\left(v\right)+f^{\prime }\left(v\right)e_{a}^{2}+o\left(e_{a}\right)\right]\approx E\left[f^{\prime }\left(v\right)\right]EMSE\right.$$

and

(35) $$E\left[f^{2}\left(e\right)\right]\approx E\left[f^{2}\left(v\right)\right]+E\left[f\left(v\right)f^{\prime \prime }\left(v\right)+{\left|f^{\prime }\left(v\right)\right|}^{2}\right]EMSE.$$

Substituting (34) and (35) into (32), one can obtain

(36) $$EMSE=\frac{E\left(X^{T}R^{-1}X\right)\cdot E\left(f^{2}\left(v\right)\right)}{2E\left(f^{\prime }\left(v\right)\right)-E\left(X^{T}R^{-1}X\right)\cdot E\left(f\left(v\right)f^{\prime \prime }\left(v\right)+f^{2}\left(v\right)\right)}$$

where $f\left(v\right)=\mu v\exp(-v^{2}/\left(2\beta^{2}\right))$, $f^{\prime }\left(v\right)=\exp\left(-v^{2}/\left(2*\beta^{2}\right)\right)-\left(v^{2}\exp\left(-v^{2}/\left(2\beta^{2}\right)\right)\right)/\beta^{2}$ and $f^{\prime \prime }\left(v\right)=\left(v^{3}\exp\left(-v^{2}/\left(2\beta^{2}\right)\right)\right)/\beta^{4}-\left(3v\exp\left(-v^{2}/\left(2\beta^{2}\right)\right)\right)/\beta^{2}$. Then, one can get the closed form solution of EMSE. The three parameters affecting the steady-state EMSE are step size μ, the noise deviation $v^{2}$ and β, the kernel width of MCC.

## 5. Experiments and Results

In this section, several experiments were carried out. In experiment 1, we simulated the algorithms in a simple system identification scenario to verify their effectiveness. Experiment 2 discussed the influence of the kernel width parameter selection of MCC-Newton under Gaussian and non-Gaussian noises. Experiment 3 compared Newtonian-type and LMS-type algorithms on the Correntropy performance surface. Experiment 4 compared the theoretical and the experimental results of EMSE of MCC-Newton. The effectiveness of MCC-Newton in practical acoustic echo cancellation was examined in experiment 5. Experiment 6 compared the echo return loss enhancement performance of different algorithms in practical acoustic echo cancellation.

**Figure 3:**
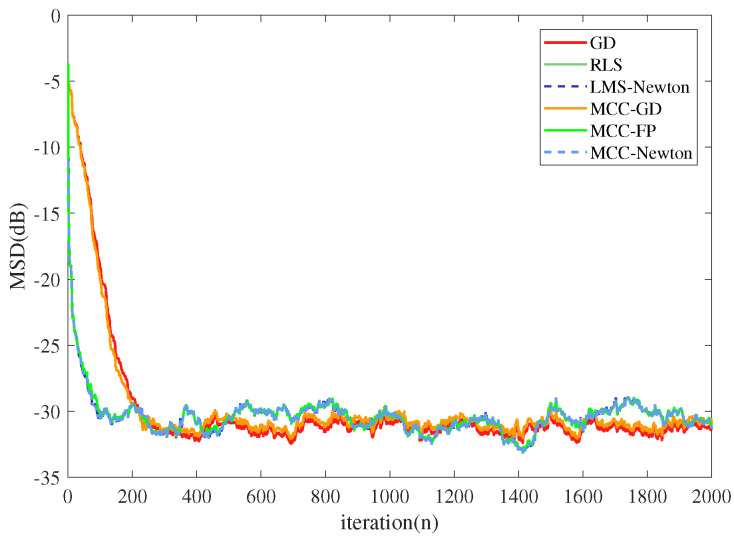
The performance of different algorithms under Gaussian noise.

**Figure 4:**
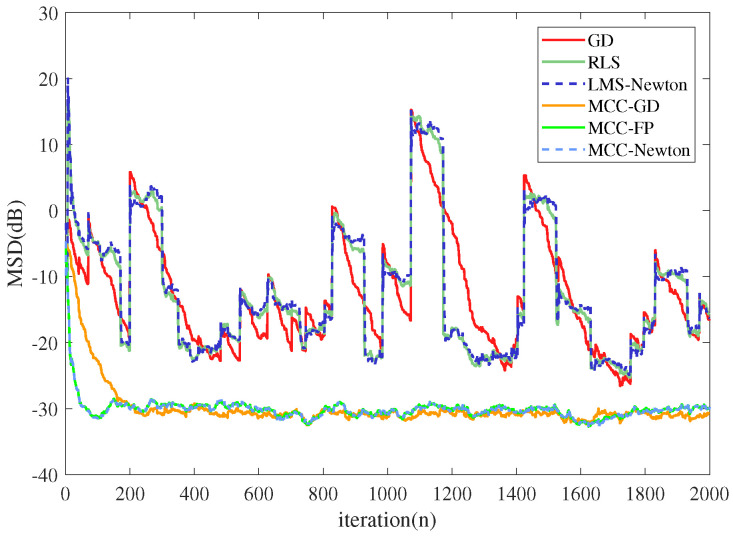
The performance of different algorithms under α stable noise.

Experiment 1:

The system impulsive response $W_{o}$ was set as a vector with 20 entries, which was consistent with the order of the adaptive filter. The 10th element of $W_{o}$ was set to 1, and the other elements were 0. The iteration number was set to 2000. The input signal followed Gaussian distribution with zero mean and unit variance. The desired signal was generated by the convolution of $W_{o}$ and the input signal, added by the system noise. We executed 100 Monte Carlo runs so that the average simulation result was available for the discussion. We simulated in both Gaussian and α stable noises, and compared two criteria (MMSE criterion and MCC)-based algorithms, each optimized with three different methods. The additive Gaussian noise had zero mean and the noise variance was 0.09. The additive α stable noise [45] was set with the same variance with the Gaussian noise, and the characteristic exponent α was set to 1.2, which is a measure of the thickness of the tails of the α stable distribution. When α is equal to 1 or 2, α stable distribution becomes the Cauchy distribution and the Gaussian distribution as special cases. To compare the convergence rate of the algorithms, step sizes were adjusted to ensure that the steady-state MSD of different algorithms were close to each other. The results are shown in Figure 3 and Figure 4.

From Figure 3 and Figure 4 one can observe that: (1) all the algorithms converge to a steady-state in the presence of Gaussian noise. However, in α stable noise environment, MMSE criterion-based algorithms could not converge to a steady-state but MCC-based algorithms still converge well. (2) Newtonian-type and RLS-type algorithms converge in similar rate, and are much faster than LMS-type algorithms.

**Figure 5:**
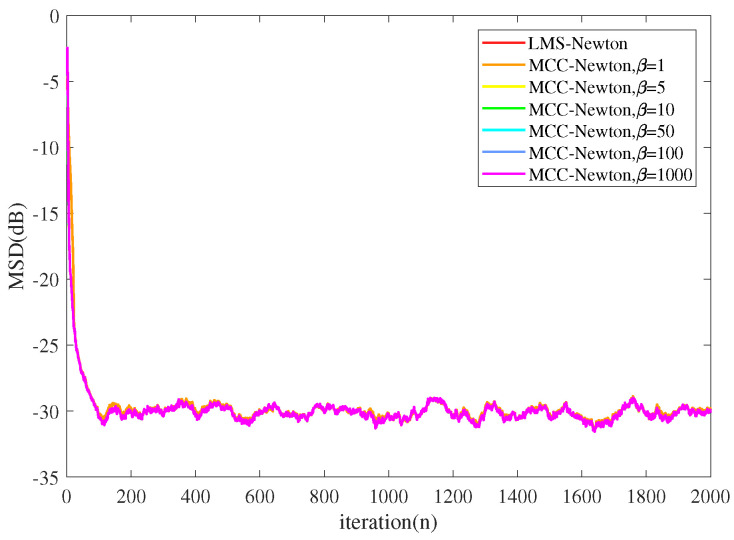
The performance of the MCC-Newton with different kernel width β under Gaussian noise.

**Figure 6:**
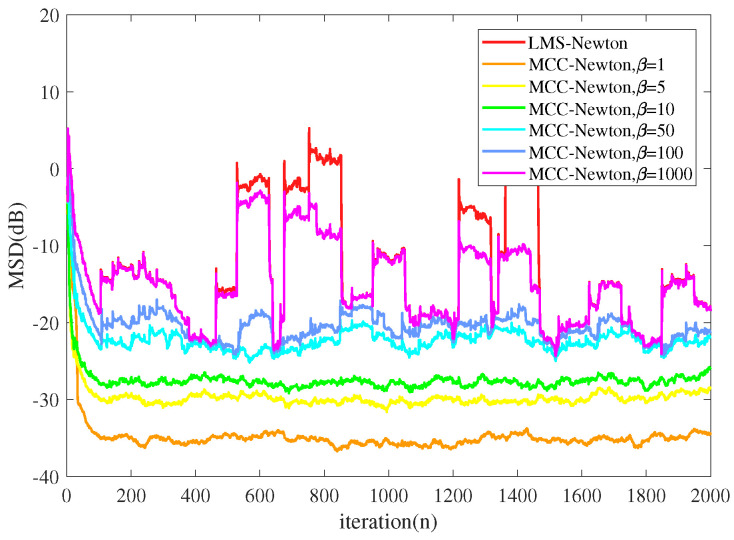
The performance of the maximum correntropy criterion (MCC)-Newton with different kernel width β under α stable noise.

Experiment 2:

We simulated the performance of MCC-Newton algorithm with different kernel widths set and the results are presented in Figure 5 and Figure 6. The parameter settings are the same as the previous experiment. From Figure 5 one can observe that under a Gaussian noise environment, the MCC-Newton algorithm performs as well as LMS-Newton algorithm, and the kernel width selection of MCC-Newton does not make much difference in terms of the steady-state MSD. However, as Figure 6 shows, LMS-Newton does not converge under the α stable noise, and MCC-Newton converges to a steady-state only when the kernel width is set small. One can also observe that MCC-Newton with a large kernel width performs similar to LMS-Newton which agrees well with the relation of the MMSE criterion and the MCC.

Experiment 3:

To image the difference of the convergence process of MCC-based Newtonian-type and LMS-type algorithms, we simulated a system identification scenario of two tap weights, and presented the weight tracks of the algorithms on the correntropy performance surface [23].

**Figure 7:**
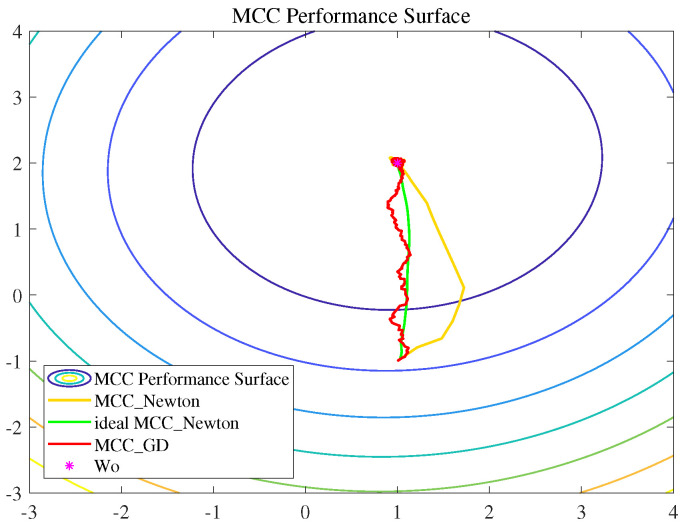
The weight tracks for the gradient descent-based algorithm (MCC_GD) and the Newtonian-type algorithm (ideal MCC_Newton and MCC_Newton) on the Correntropy performance surface.

The system impulsive response was set as [1,2], and the initial one was [1,−1]. Figure 7 shows the weight tracks (i.e., how the impulsive response W changes to approach the optimal one $W_{o}$) of different algorithms. One can observe that there are many twists and turns in the curve of MCC_GD than MCC_Newton, so that MCC_GD needs much more steps to reach an estimation close to $W_{o}$, which is consistent with one of the results of experiment 1; that Newtonian-type algorithms converge faster than LMS-type ones. Figure 7 also shows that the ideal MCC_Newton algorithm possesses the best direction of optimization and the weight track of MCC_GD is approximately consistent with the gradient descent. However, it seems hard for the practical MCC_Newton algorithm to find the right direction at the beginning of the iteration. This can be explained by the temporary imprecision of the correlation matrix estimation in the practical MCC_Newton. The difference between the ideal and the practical MCC_Newton algorithms is the calculation of the correlation matrix of the input signal. The ideal MCC_Newton algorithm can use the perfectly calculated correlation matrix through the whole iteration, but the practical one can only build the estimated correlation matrix from the received input signals, which causes the imprecision of the estimation of the matrix at the beginning of the iteration. When the number of iterations increases, the estimation becomes more accurate, so that the practical MCC-Newton algorithm will achieve a similar performance with the ideal one after a certain number of iterations.

**Figure 8:**
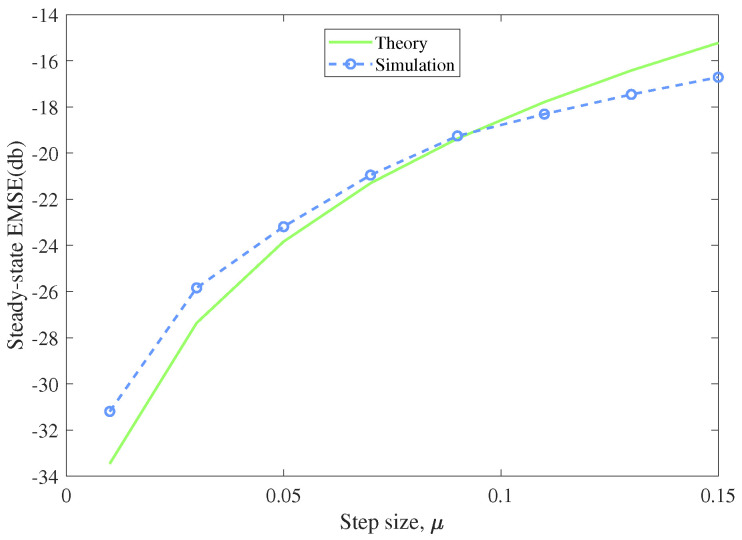
Theoretical and simulated EMSEs versus the step size μ $\left(\beta =5,\sigma_{v}^{2}=0.09\right)$.

**Figure 9:**
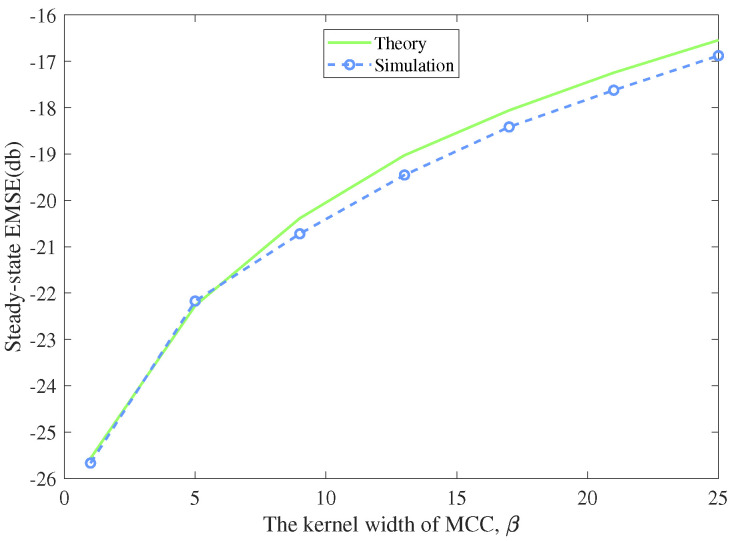
Theoretical and simulated excess mean square errors (EMSEs) versus the kernel width β $\left(\mu =0.07,\sigma_{v}^{2}=0.09\right)$.

**Figure 10:**
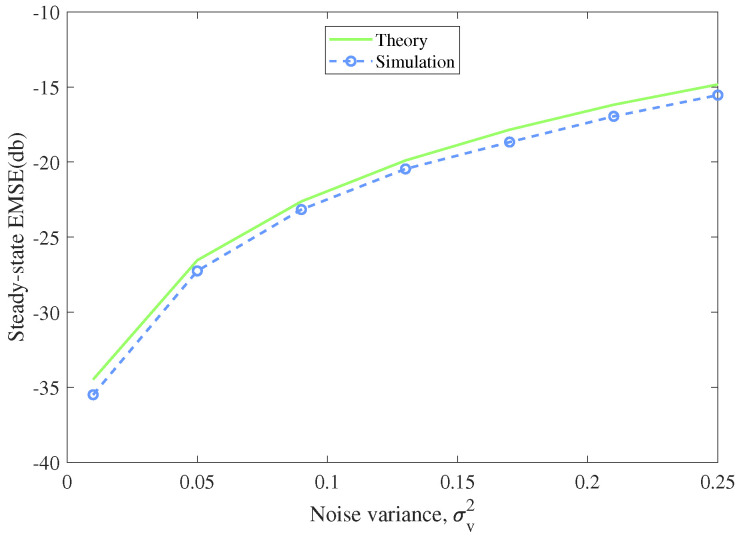
Theoretical and simulated EMSEs versus the noise variance $\sigma_{v}^{2}$ (μ=0.07,β=5).

Experiment 4:

In this experiment, we compared the theoretical derived EMSE in Section 4 with the simulated one to confirm the theoretical result. The input signal was generated from a Gaussian distribution with zero mean and unit variance, and the additive noise was Gaussian distributed with specified variance. We executed 100 Monte Carlo runs to get the average simulation result of EMSE. From (36), we can learn that the steady-state EMSE are determined by step size μ, the noise deviation $v^{2}$ and β, the kernel width of MCC. So we separately simulated the steady-state performance versus different parameters. In each simulation, one parameter varied with the other two parameters stayed unchanged, so we could display the influence of the specified parameter to the steady-state EMSE in Figure 8, Figure 9 and Figure 10.

Figure 8 represents the theoretical and simulated EMSEs under different step sizes. The kernel width β was set to 5, and the noise variance $\sigma_{v}^{2}$ was set to 0.09. The step size μ scaled from 0.01 to 0.15. Figure 9 shows the theoretical and simulated EMSEs versus different kernel widths which scaled from 1 to 25. The step size was 0.07 and the noise variance was set as 0.09. Figure 10 displays the theoretical and simulated EMSEs under noise variances scaling from 0.01 to 0.25. The step size was 0.07 and the kernel width was set to 5. One could observe that the simulation results agrees well with the theoretical analysis. The steady-state performance of the algorithm degrades with the increase of the step size, the kernel width and the noise variance. In other words, we can choose smaller step size and kernel width to help MCC-Newton algorithm achieve better steady-state performance.

Experiment 5:

We examined the algorithm in practical acoustic echo cancellation and the parameters in this experiment were set as follows. We recorded two different simple voices that both last for 4.0 s as the near end speech and the far end speech. In ideal acoustic echo cancellation, the echo caused by the far end speech should be well cancelled from the mixed speech picked up by the near end microphone thus the output of the canceller could be close to the near end speech. The size of the impulsive response $W_{o}$ was set to 2000, which was large enough so that the generated echo can be heard clearly.

To evaluate the performance in AEC, we introduce the echo return loss enhancement (ERLE) which is defined as:

(37) $$\mathrm{ERLE}=\frac{E\left\{{\left(d(n)-\epsilon (n)\right)}^{2}\right\}}{E\left\{{\left(e(n)-\epsilon (n)\right)}^{2}\right\}},$$

where d(n) is the echo signal of the far end speech picked up by the microphone, which contains the additive noise ϵ(n) and the original echo signal. e(n) is the residual error of AEC which is transmitted back to the far end together with the near end speech. So, ERLE tells us how large the estimated echo is compared with the residual error of AEC, without the influence of the additive noise.

**Figure 11:**
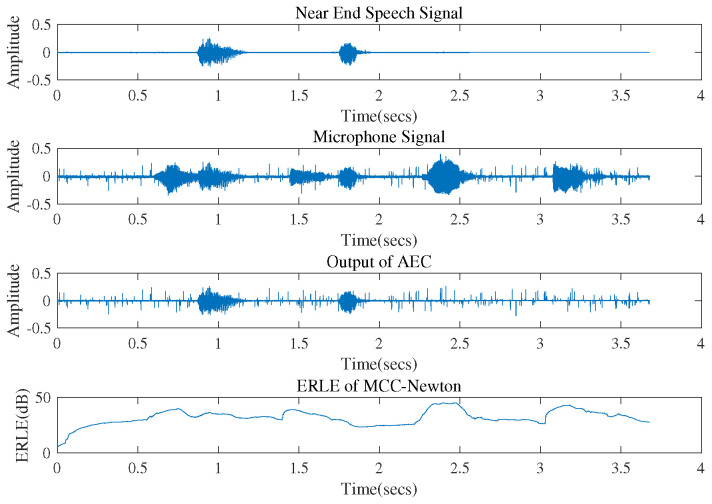
Effect of the MCC-Newton algorithm and the echo return loss enhancement performance.

We used the MCC-Newton algorithm in practical acoustic echo cancellation where the additive noise picked up by the near end microphone follows non-Gaussian distribution. To focus on the problem of AEC, it was assumed that we already had an ideal detection scheme to tell when near end speech was present. Figure 11 shows the practical effect and the ERLE performance of MCC-Newton. One can observe that: (1) the microphone signal contains the near end speech, the far end speech and its echo signal added with the system noise. (2) The AEC output is free from the far-end speech echo and is very close to the original near-end speech. However, it could not get rid of the influence of noise. (3) ERLE becomes larger when there exists echo signal of the far end speech. The algorithm could achieve up to 50 dB ERLE.

**Figure 12:**
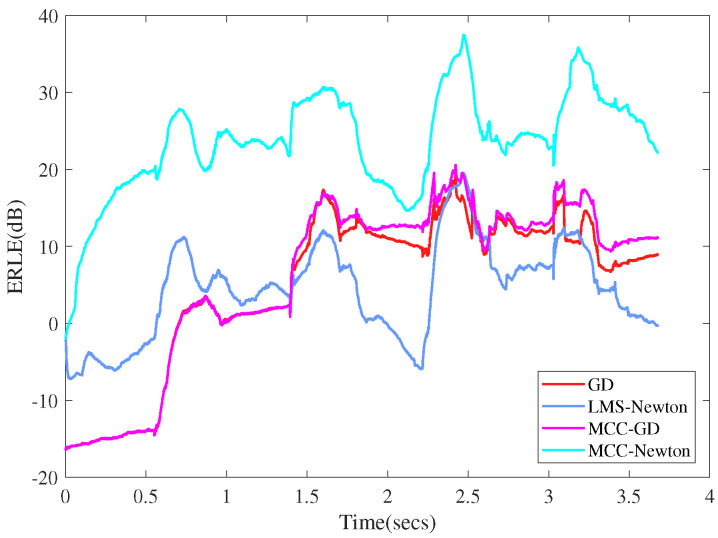
Echo return loss enhancement (ERLE) of different algorithms.

Experiment 6:

We compared the ERLEs of MCC-Newton algorithms with that of LMS-Newton and the conventional LMS-type algorithms to see their performance under practical AEC scenario. The parameter settings are similar with that of the previous experiment. Figure 12 shows the result, from which one can observe that: (1) the ERLEs of all the algorithms tends to increase at the early stage of the experiment, and the ERLEs of Newtonian-type algorithms increase faster than that of LMS-type algorithms. The possible explanation is that the algorithms need to converge to a steady-state in the early stage iterations and Newtonian-type algorithms converge faster than LMS-type ones. (2) Compared with the other algorithms, MCC-Newton achieves higher ERLE performance. This confirms the practical effectiveness of MCC-Newton algorithm in the presence of heavy tailed mixed Gaussian noise.

## 6. Conclusions

MCC is recently adopted to deal with the heavy tailed impulsive noise problem in robust adaptive filtering. In this paper, a Newtonian-type method is innovatively used to solve the MCC-based adaptive filtering, of which the existing optimization methods are usually LMS-type or RLS-type. Experiments demonstrate that the Newtonian-type MCC-based adaptive filtering converges as fast as RLS-type ones, and is much faster than LMS-type. The steady-state performance of the Newtonian-type MCC adaptive filtering is theoretically analyzed and is verified to be consistent with the experiment results. It is also revealed in experiments that a smaller kernel width helps the Newtonian-type MCC-based adaptive filtering perform better under impulsive noises, which could be a guide for the selection of the parameter. Experiments also show that the MCC-Newton algorithm is practical in acoustic echo cancellation under heavy-tailed system noise. In future work, better approximation methods of the Hessian matrix could be involved to decrease the computational complexity and further improve the performance of the algorithm.
